# Metabolic regulatory mechanisms of Yijinjing exercise in patients with type 2 diabetes mellitus: Insight from the gut microbiota-intestinal barrier- inflammation axis

**DOI:** 10.3389/fendo.2026.1848478

**Published:** 2026-06-24

**Authors:** Min Li, Xinhai Yang, Yali Wen

**Affiliations:** 1 College of Physical Education and Health Sciences, Chengdu College of Arts and Sciences, Chengdu, Sichuan, China; 2 College of Physical Education and Health Sciences, Sichuan Technology and Business University, Chengdu, Sichuan, China

**Keywords:** glucose homeostasis, gut microbiota, inflammatory cytokines, intestinal mucosal barrier function, type 2 diabetes mellitus, Yijinjing exercise

## Abstract

**Objective:**

This study aimed to explore the impact of Yijinjing exercise on glucose metabolic homeostasis, systemic inflammatory markers, and the composition of gut microbiota in individuals diagnosed with type 2 diabetes mellitus (T2DM).

**Methods:**

A total of 45 T2DM patients participated in a 6-month structured Yijinjing exercise program. Body composition metrics were evaluated via bioelectrical impedance analysis. Standard biochemical indices, such as fasting insulin, blood glucose, lipid profiles (total cholesterol, triglycerides, and high/low-density lipoprotein cholesterol), and glycated hemoglobin (HbA1c), were quantified using automated laboratory analyzers. Serum concentrations of inflammatory cytokines (TNF-α, IL-6, IL-1β, IL-10, CRP), intestinal barrier permeability markers (D-lactate and Zonulin), and the mucosal repair factor MFG-E8 were determined through enzyme-linked immunosorbent assay (ELISA). Furthermore, the gut microbial community structure was profiled by 16S rRNA gene sequencing.

**Results:**

Following the 6-month intervention, participants demonstrated a significant improvement in body composition, characterized by reductions in body weight, BMI, waist circumference, and body fat percentage, coupled with an increase in lean mass (P < 0.05). Metabolic and inflammatory profiles showed notable improvements, with decreased levels of fasting blood glucose, HbA1c, HOMA-IR, CRP, TNF-α, IL-6, IL-1β, IL-8, and total cholesterol, while the anti-inflammatory cytokine IL-10 was significantly upregulated (P < 0.01). Ecological analysis of the gut microbiota indicated an increase in both Chao1 and Shannon diversity indices (P < 0.05). Specifically, the abundance of beneficial taxa, such as Lactobacillus and Bifidobacterium, was markedly elevated; conversely, potential pathogens including Escherichia coli, Klebsiella pneumoniae, Desulfovibrio, and Candida albicans were significantly suppressed (P < 0.01). Furthermore, the intervention mitigated intestinal mucosal damage, as evidenced by the downregulation of D-LA and Zonulin and the upregulation of MFG-E8 (P < 0.01).

**Conclusion:**

T2DM is associated with gut dysbiosis, compromised intestinal barrier integrity, and chronic systemic inflammation. Yijinjing exercise serves as an effective intervention to optimize glucose control, restore microbial diversity, fortify the intestinal mucosal barrier, and suppress systemic inflammation. These improvements occurred concurrently with significant remodeling of the gut microbiota, intestinal barrier restoration, and resolution of systemic inflammation, suggesting that gut microbiota modulation may have contributed, at least in part, to the observed metabolic benefits. These results suggest that Yijinjing exercise, as a non-pharmacological approach associated with favorable gut microbiota adaptations, may represent a valuable and personalized strategy for T2DM management, though further studies are warranted to establish the directionality and independence of these interrelated pathways.

## 1 Introduction

Type 2 diabetes mellitus (T2DM) has emerged as a formidable global public health crisis, with the global patient population projected to reach nearly 700 million by 2045 (1). As a complex metabolic disorder, T2DM is driven not only by genetic predisposition but also by environmental determinants, with the interplay between insulin resistance and chronic low-grade inflammation serving as a focal point of recent research (2–4). Increasingly, the gut microbiota is recognized as a pivotal “hub” mediating the crosstalk between environmental factors and host metabolism (5). Dysbiosis of the gut microbiota can compromise intestinal barrier integrity—a phenomenon often termed “leaky gut”—facilitating the translocation of bacterial products and triggering systemic inflammation, which in turn exacerbates insulin resistance and metabolic dysfunction (6).

While the clinical benefits of aerobic exercise in diabetes management are well-established, primarily attributed to enhanced skeletal muscle glucose uptake and cardiovascular health (7), the regulatory impact of exercise on the gut microbiome—the “second genome”—remains a burgeoning area of investigation. Emerging evidence suggests that different modalities of exercise may differentially influence gut microbial composition and function. Conventional aerobic exercise has been shown to increase the relative abundance of short-chain fatty acid (SCFA)-producing bacteria, such as Faecalibacterium prausnitzii and Roseburia, and to enhance microbial diversity in both healthy individuals and metabolically compromised populations (8, 9). However, conventional exercise interventions predominantly activate skeletal muscle metabolic pathways and induce cardiovascular adaptations, with comparatively limited effects on neuroimmune signaling and autonomic nervous system modulation—pathways increasingly recognized as key regulators of the gut microenvironment.

In contrast, mind-body exercises—a category encompassing Tai Chi, Qigong, yoga, and related practices—integrate coordinated breathing, meditative focus, and deliberate movement, thereby engaging both the somatic and autonomic nervous systems simultaneously. Recent investigations have begun to reveal the distinct gut microbiota-modulating potential of these practices. For instance, Tai Chi practice has been associated with increased abundances of Lactobacillus and Bifidobacterium and reduced levels of pro-inflammatory cytokines in older adults (10). Similarly, yoga-based interventions have been reported to favorably shift gut microbial composition, with notable increases in commensal bacteria and concurrent reductions in intestinal permeability markers (11). Mechanistically, these effects have been partially attributed to vagal nerve activation induced by diaphragmatic breathing, which modulates intestinal motility and mucosal immune function via the gut-brain axis, as well as to attenuation of hypothalamic-pituitary- adrenal (HPA) axis hyperactivation, which is known to compromise intestinal barrier integrity under chronic psychological stress (12). Collectively, these findings suggest that mind-body exercises may engage the gut-immune-metabolic axis through pathways that are not fully replicated by conventional unimodal aerobic exercise.

Crucially, the mechanism by which exercise modulates the “gut- immune -metabolic” axis to restore metabolic homeostasis is not yet fully elucidated. Yijinjing, a traditional Chinese mind-body practice characterized by rhythmic stretching, diaphragmatic breathing, and meditative focus, offers a unique physiological stimulus (13). As one of the oldest and most systematically codified forms of Chinese mind-body exercise, Yijinjing shares mechanistic features with Tai Chi and Qigong - including parasympathetic activation and whole-body sequential movement- while incorporating a distinctive emphasis on sustained musculotendinous stretching and breath-movement coordination that may provide additional physiological stimuli relevant to gut microbiota modulation and intestinal barrier maintenance. Despite its long history of clinical application in metabolic and inflammatory conditions, evidence regarding its efficacy in modulating the gut-microbiota-mucosal barrier axis in T2DM remains limited, and no prior study has systematically investigated the interplay among gut microbial remodeling, intestinal barrier function, and systemic inflammation in the context of Yijinjing intervention. Therefore, this study aims to systematically evaluate the impact of a 6-month Yijinjing intervention on glucose homeostasis, systemic inflammation, gut microbial composition, and intestinal mucosal barrier function in T2DM patients. By elucidating the underlying biological mechanisms, this research seeks to provide a robust scientific rationale for integrating traditional mind-body exercise into contemporary non-pharmacological diabetes management.

## 2 Subjects and methods

### 2.1 Ethical statement and study design

This investigation utilized a longitudinal, self-controlled, pre-post design. The research protocol received ethical clearance from the Institutional Review Board of Chengdu College of Arts and Sciences (Ref: CDUN-2024-00103) and strictly adhered to the Declaration of Helsinki. All participants provided written informed consent prior to their inclusion in the study. To maintain participant confidentiality and data integrity, all identifiable personal information was anonymized and replaced with unique alphanumeric identifiers (e.g., T2D-001). Furthermore, all research datasets were maintained in a secure, password-encrypted database, with access restricted exclusively to the authorized research team.

### 2.2 Study participants

A total of 107 patients with type 2 diabetes mellitus (T2DM) admitted to the Sichuan Provincial People’s Hospital were initially screened. To minimize potential confounding factors, 45 male T2DM patients were enrolled in this study (mean age: 52.78 ± 6.34 years). All participants were non-smokers and non-drinkers. The inclusion criteria were as follows: (1) diagnosis of T2DM in accordance with the Guidelines for the Prevention and Treatment of Type 2 Diabetes in China (2013 Edition); (2) T2DM diagnosed at least 2 years prior to enrollment, without the requirement for insulin therapy; (3) absence of diabetes-specific complications or ischemic heart disease; and (4) the ability to perform physical activities. The exclusion criteria were: (1) presence of endocrine disorders, inflammatory bowel diseases, or malabsorption syndromes; (2) history of smoking, alcohol consumption, or substance abuse; and (3) use of antibiotics, steroids, laxatives, antidiarrheals, or probiotics within the 3 months prior to or during the study period. Written informed consent was obtained from all participants and their families. No participants withdrew from the study during the 6-month intervention period, resulting in a retention rate of 100% (45/45). Exercise compliance was monitored continuously via attendance records maintained by the supervising instructor. All participants completed the full intervention protocol and demonstrated consistently high adherence throughout the study duration. No adverse events related to the Yijinjing exercise program were reported.

### 2.3 Methods

#### 2.3.1 Anthropometric measurements

Height, body weight, body mass index (BMI), and waist circumference were measured using standardized protocols. Body composition metrics, specifically body fat mass and lean body mass, were quantified via bioelectrical impedance analysis (BIA).

#### 2.3.2 Biochemical and immunological assays

Fasting insulin, fasting blood glucose (FBG), total cholesterol (TC), triglycerides (TG), as well as low-density and high-density lipoprotein cholesterol (LDL-C and HDL-C) were analyzed using an automated biochemical analyzer. Glycated hemoglobin (HbA1c) levels were determined through an automated hemoglobin analyzer. Serum concentrations of inflammatory cytokines (TNF-α, IL-6, IL-1β, IL-10, and CRP), intestinal mucosal permeability markers (D-lactate and Zonulin), and the mucosal repair protein MFG-E8 were evaluated utilizing enzyme-linked immunosorbent assay (ELISA) kits.

#### 2.3.3 Gut microbiota profiling

Fecal samples were collected from each participant. Total genomic DNA was extracted following the instructions of the DNA extraction kit. The V3 hypervariable region of the 16S rRNA gene was amplified using real-time quantitative PCR. Sequencing was performed on the Illumina MiSeq platform. The resulting sequences were aligned against the Silva database, and sequences sharing >97% similarity were clustered into operational taxonomic units (OTUs). Alpha diversity indices (e.g., Chao1 and Shannon) were calculated based on the OTU clusters to evaluate changes in gut microbial richness, diversity, and community structure.

### 2.4 Intervention protocol

#### 2.4.1 Yijinjing exercise program

Participants engaged in the standardized Yijinjing exercise program. Prior to enrollment, all subjects underwent a comprehensive safety assessment by professional exercise therapists. Participants were grouped based on their residential proximity and personal preferences, with a designated team leader responsible for monitoring attendance, exercise adherence, and dietary consistency. During the first 4 weeks, subjects received intensive training from certified instructors to ensure technical proficiency in all 12 movements. From the 5th week onward, team leaders supervised group sessions in local community settings. The intervention lasted for 6 months, consisting of 5 sessions per week, with each session comprising 3 repetitions of the 12-movement Yijinjing sequence (totaling 45–60 minutes per session, based on the 15–20 minute duration per full sequence). The protocol followed the official 2004 version promoted by the General Administration of Sport of China. Researchers conducted daily monitoring to ensure strict compliance with the exercise regimen.

#### 2.4.2 Characteristics of Yijinjing and comparison with other exercise modalities

Yijinjing is a traditional Chinese mind-body exercise with a history of over a thousand years, involving slow, coordinated whole-body movements integrated with deep diaphragmatic breathing and meditative focus. In terms of exercise intensity, Yijinjing is generally classified as light-to-moderate intensity (approximately 2–4 METs). Each of the 12 movements is performed in a continuous, flowing manner with breath coordination, targeting specific muscle groups, tendons, and fascial chains throughout the body.

Compared with conventional moderate-intensity aerobic exercise (e.g., brisk walking or cycling), Yijinjing is characterized by lower cardiovascular demand, greater emphasis on postural alignment, dynamic muscle stretching, and neuromuscular coordination, with deliberate integration of breathing regulation throughout each movement. Relative to other mind-body interventions, Yijinjing shares similarities with Tai Chi in terms of exercise intensity and movement fluidity, but places particular emphasis on dynamic stretching of the tendons and fascia, as reflected in its name (“Yi Jin,” meaning “transforming the tendons”). Unlike yoga, which emphasizes static posture holding, Yijinjing incorporates continuous transitions between movements guided by breath control, making it more accessible for older adults and individuals with chronic metabolic conditions such as T2DM. Compared with Qigong, which primarily focuses on breathing regulation and mental visualization with minimal physical movement, Yijinjing involves a greater degree of whole-body dynamic movement and musculoskeletal engagement. These distinguishing characteristics suggest that the metabolic and gut microbiota-related benefits observed in the present study may reflect the combined influence of reduced systemic stress responses, enhanced parasympathetic tone, improved gut motility, and musculoskeletal activation associated with this unique form of mind-body practice.

### 2.5 Dietary control

To minimize the potential confounding influence of dietary changes on gut microbiota composition and metabolic outcomes, all participants were explicitly instructed to maintain their habitual dietary patterns throughout the 6-month intervention period. Participants were advised not to modify their usual energy intake, macronutrient composition, or food preferences during the study. Dietary adherence was monitored using structured 3-day dietary records (two weekdays and one weekend day) collected at multiple time points across the intervention period. The records were reviewed and analyzed by trained researchers to assess the stability of dietary habits over time and to identify any notable deviations from habitual intake. Participants who reported substantial dietary changes were followed up individually to reinforce dietary consistency. The designated team leaders were additionally responsible for reminding participants of dietary requirements during each supervised exercise session, thereby reinforcing adherence through regular face-to-face contact.

### 2.6 Statistical analysis

Data were analyzed using SPSS 19.0 (SPSS Inc., Chicago, IL, USA), and graphs were generated using GraphPad Prism 8.0 (GraphPad Software, San Diego, CA, USA). Continuous variables are expressed as mean ± standard deviation (X ± S) for normally distributed data, or median (interquartile range, IQR) for non-normally distributed data. For group comparisons, normally distributed data were analyzed using one-way analysis of variance (ANOVA) followed by *post-hoc* tests (e.g., Tukey’s test), while non-normally distributed data were analyzed using the Kruskal-Wallis H test (or Mann-Whitney U test for two-group comparisons). Correlations were assessed using Pearson’s correlation coefficient for normally distributed data and Spearman’s rank correlation for non-normally distributed data. The strength of correlation was interpreted as follows: r < 0.4 (low), 0.4 ≤ r ≤ 0.7 (moderate), and r > 0.7 (high). To examine whether the associations between key microbial taxa and metabolic/inflammatory indices were independent of body composition changes, partial correlation analyzes were performed controlling for BMI and body fat percentage, using Pearson’s or Spearman’s partial correlation as appropriate based on the distributional properties of the variables. A two-sided P < 0.05 was considered statistically significant. No formal adjustment for multiple comparisons was applied to the primary hypothesis-driven analyzes involving pre-specified clinical outcome variables. For secondary and exploratory analyzes involving multiple microbial taxa and intestinal barrier indices, results should be interpreted with appropriate caution given the potential for inflated type I error in the context of multiple simultaneous comparisons.

## 3 Result

### 3.1 Anthropometric and body composition measurements

**Figure 1:**
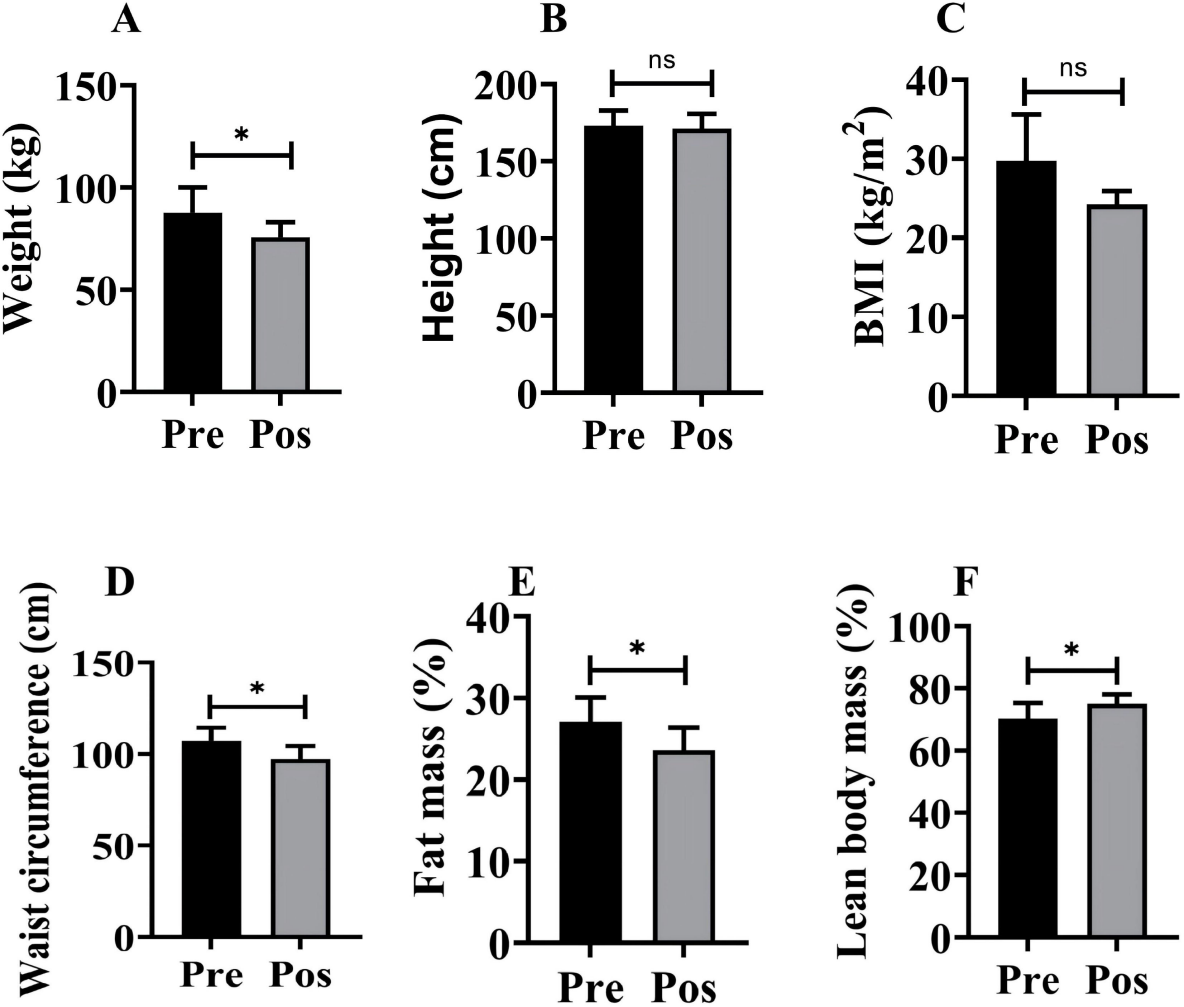
Changes in anthropometric and body composition metrics following Yijinjing exercise. Pre: before exercise; Pos: after 6 months of exercise. ns, no significant. Compared with Pre, *P< 0.05.

Significant modifications in body composition were observed after the 6-month exercise program. Compared with pre-intervention values, T2DM patients demonstrated substantial decreases in body weight, waist circumference, and body fat percentage, while lean body mass was significantly elevated (P < 0.05) (**Figure 1**).

### 3.2 Changes in biochemical and inflammatory indices

**Figure 2:**
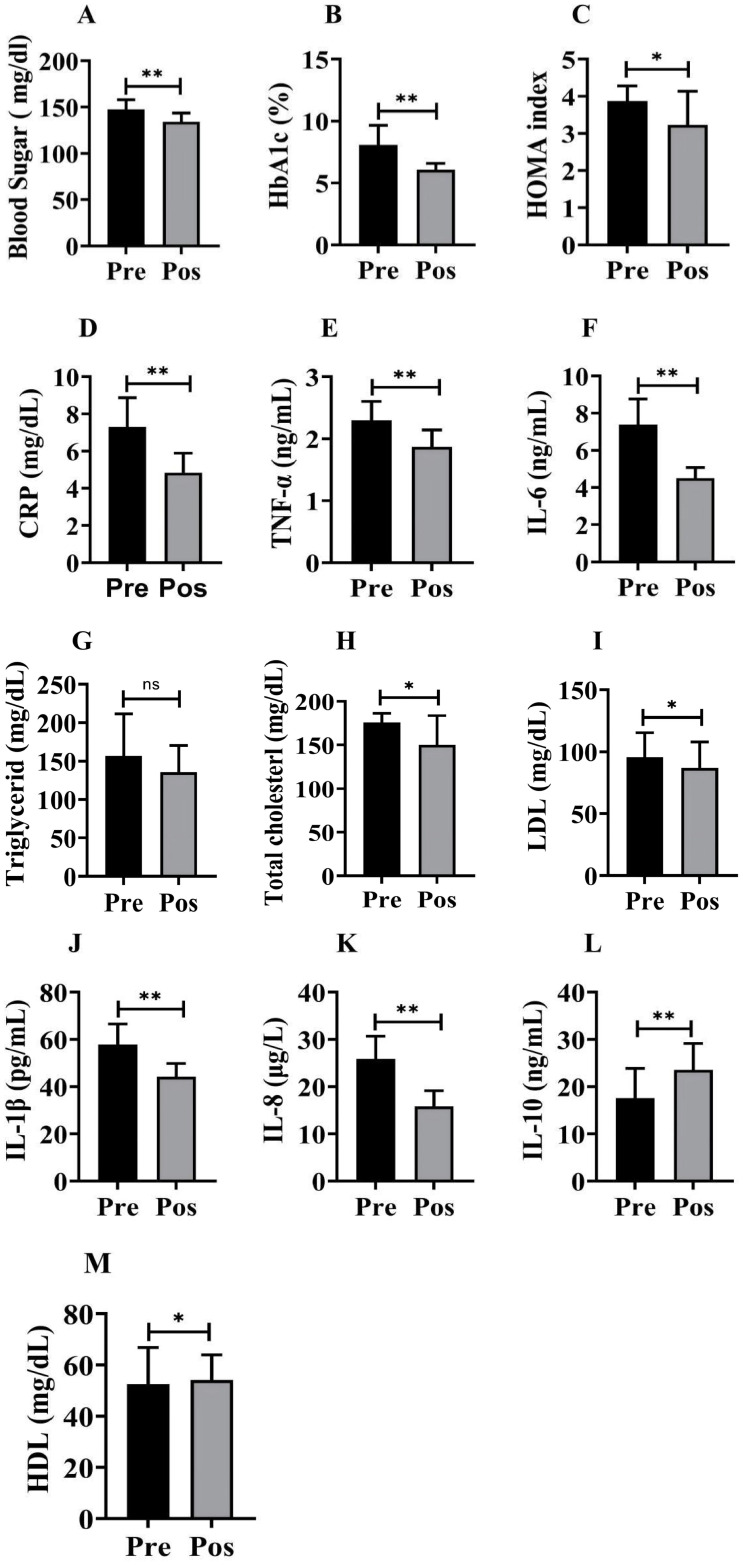
Changes in metabolic, inflammatory, and insulin resistance indicators before and after the 6-month Yijinjing intervention. Pre, before exercise; Pos, after 6 months of exercise. ns, no significant. Compared with Pre, *P< 0.05; **P< 0.01.

Following the 6-month Yijinjing program, patients exhibited a favorable shift in metabolic and inflammatory status. Fasting blood glucose, glycated hemoglobin (HbA1c), homeostasis model assessment of insulin resistance (HOMA-IR), C-reactive protein (CRP), tumor necrosis factor-alpha (TNF-α), interleukin-6 (IL-6), interleukin-1 beta (IL-1β), interleukin-8 (IL-8), low-density lipoprotein cholesterol (LDL-C), and total cholesterol were significantly reduced (P < 0.01, P < 0.05), whereas interleukin-10 (IL-10) and high-density lipoprotein cholesterol (HDL-C) levels were significantly increased compared to pre-intervention values (P < 0.01, P < 0.05) (**Figure 2**).

### 3.3 Gut microbiota compositional changes

**Figure 3:**
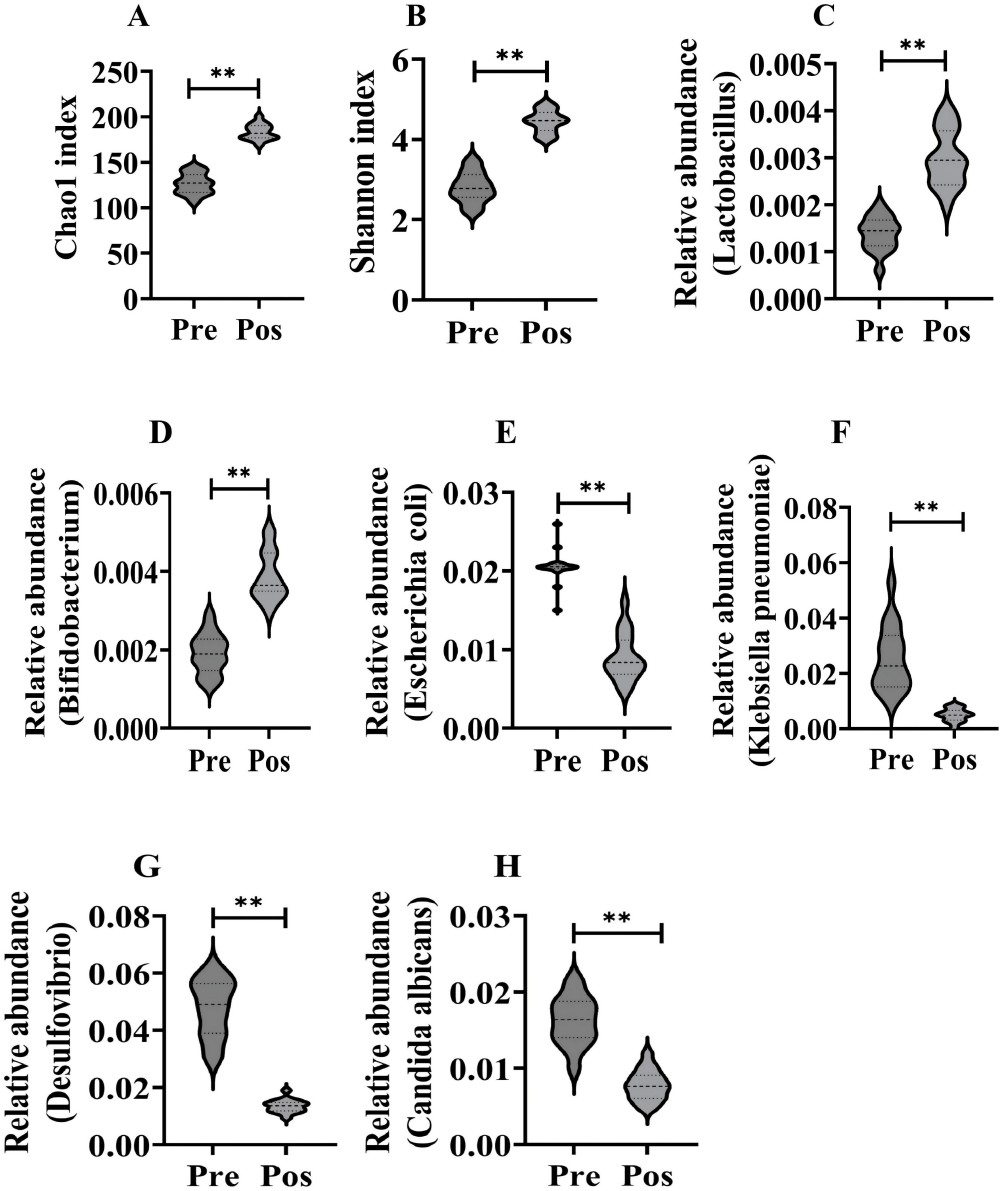
Alterations in gut microbiota diversity and composition following the 6- month Yijinjing intervention. **(A, B)** Alpha diversity indices (Chao1 and Shannon). **(C–H)** Relative abundances of significantly altered bacterial genera. Pre, before exercise; Pos, after 6 months of exercise. Compared with Pre, **P< 0.01.

Following the 6-month Yijinjing intervention, the alpha diversity of the gut microbiota was significantly enhanced in T2DM patients, as evidenced by marked increases in both Chao1 and Shannon indices (P < 0.01). At the genus level, the relative abundances of beneficial taxa, specifically Lactobacillus and Bifidobacterium, were significantly upregulated. In contrast, the abundances of potential pathogens, including Escherichia coli, Klebsiella pneumoniae, Desulfovibrio, and Candida albicans, were significantly attenuated (P < 0.01) (**Figures 3A–H**).

### 3.4 Changes in intestinal mucosal barrier markers

**Figure 4:**
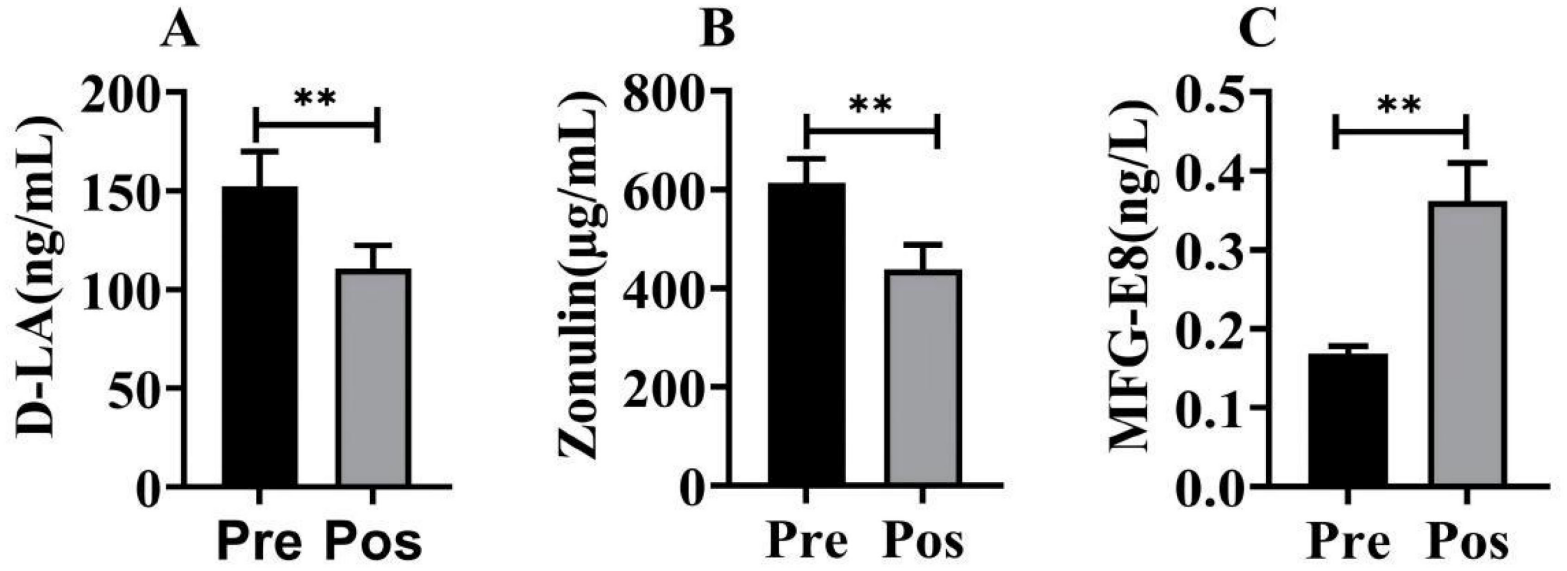
Impact of Yijinjing exercise on intestinal mucosal barrier function markers. **(A)** Serum D-lactate (D-LA) levels; **(B)** Serum Zonulin levels; **(C)** Serum MFG-E8 levels. Pre, before exercise; Pos, after 6 months of exercise. Compared with Pre,**P< 0.01.

Post-intervention assessment revealed that the 6-month Yijinjing exercise program significantly modulated markers of intestinal mucosal barrier integrity. Specifically, serum levels of D-lactate (D-LA) and Zonulin were markedly downregulated, while the mucosal repair factor milk fat globule-EGF factor 8 (MFG-E8) was significantly upregulated compared with baseline levels (P < 0.01) (**Figures 4A–C**).

### 3.5 Correlation analysis between gut microbiota, inflammatory cytokines, and intestinal barrier markers

**Figure 5:**
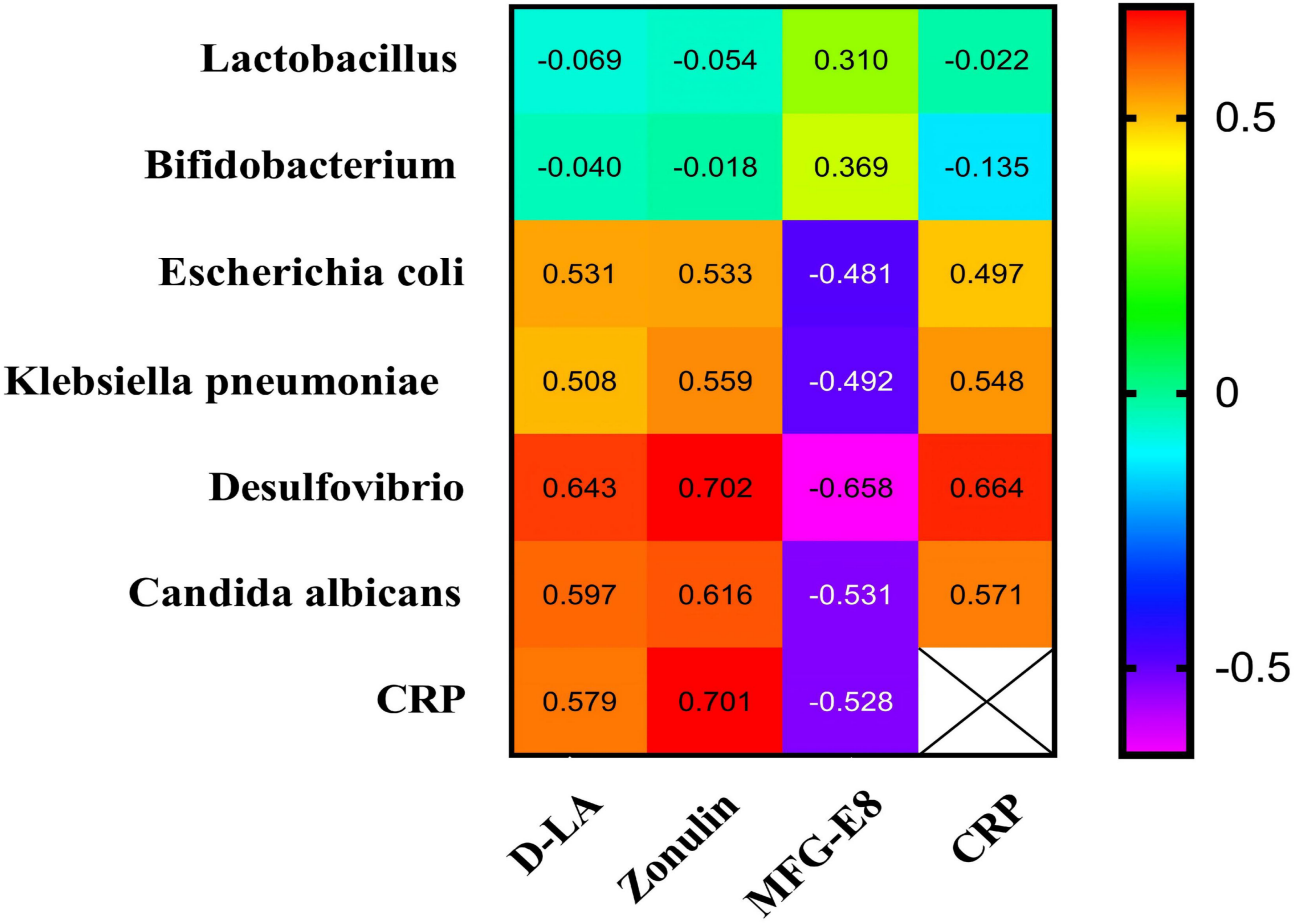
Interrelationships among gut microbiota composition, systemic inflammatory markers, and intestinal barrier integrity. Color-coded correlation coefficients represent the strength and direction of the associations (P < 0.01).

Spearman’s rank correlation analysis revealed significant associations between the gut microbiota and host metabolic/inflammatory profiles. The relative abundances of Escherichia coli, Klebsiella pneumoniae, Desulfovibrio, and Candida albicans were positively correlated with the inflammatory marker CRP and intestinal permeability markers (D-LA and Zonulin), but negatively correlated with the mucosal repair protein MFG-E8 (P < 0.01). Furthermore, CRP levels exhibited a significant positive correlation with D-LA and Zonulin, whereas a significant negative correlation was observed between CRP and MFG-E8 (P < 0.01) (**Figure 5**).

### 3.6 Partial correlations between changes in gut microbiota composition and metabolic/inflammatory indices

To examine whether the associations between gut microbiota changes and metabolic/inflammatory outcomes were independent of body composition alterations, partial correlation analyzes were performed with BMI and body fat percentage as control variables. After controlling for body composition changes, the increases in beneficial taxa—Lactobacillus and Bifidobacterium—remained significantly and negatively correlated with HOMA-IR, FBG, HbA1c, CRP, and IL-6 (all P < 0.05). Conversely, the increases in potentially pathogenic taxa—Escherichia coli, Klebsiella pneumoniae, Desulfovibrio, and Candida albicans—remained significantly and positively correlated with the same metabolic and inflammatory indices (all P < 0.05). These results suggest that the gut microbiota remodeling induced by Yijinjing exercise contributed to improvements in insulin resistance, glycemic control, and systemic inflammation independently of the concurrent body composition changes (**Table 1**).

**Table 1:** Partial correlations between changes in gut microbiota composition and metabolic/inflammatory indices.

Microorganism	HOMA-IR	FBG	HbA1c	CRP	IL-6
Lactobacillus	r=−0.52*	r=−0.48*	r=−0.45*	r=−0.56**	r=−0.51*
Bifidobacterium	r=−0.49*	r=−0.46*	r=−0.43*	r=−0.53**	r=−0.47*
Escherichia coli	r=0.58**	r=0.54**	r=0.51*	r=0.61**	r=0.57**
Klebsiella pneumoniae	r=0.54**	r=0.5*	r=0.47*	r=0.58**	r=0.53**
Desulfovibrio	r=-0.51*	r=0.47*	r=0.44*	r=0.55**	r=0.52*
Candida albicans	r=0.48*	r=0.44*	r=0.42*	r=0.50*	r=0.46*

*p<0.05, **p<0.01. Controlling variables:BMI and body fat.

## 4 Discussion

Type 2 diabetes mellitus (T2DM) is characterized not only by impaired glucose metabolism but also by profound gut dysbiosis, characterized by reduced microbial diversity, an overabundance of pathobionts, and a depletion of beneficial commensals (14). These microbial shifts are closely linked to compromised intestinal mucosal barrier integrity and chronic systemic inflammation, which collectively exacerbate insulin resistance and metabolic dysfunction (6). In this study, we demonstrate that a 6-month Yijinjing exercise intervention significantly reverses these pathological alterations. Our findings provide empirical evidence that Yijinjing exercise effectively restores gut microbial richness and diversity, suppresses the abundance of pro-inflammatory pathobionts, and reinforces the intestinal mucosal barrier, and these changes occurred concurrently with improvements in glucose homeostasis, insulin sensitivity, and systemic inflammation. Partial correlation analyzes further revealed that the associations between key microbial taxa and metabolic/inflammatory indices remained statistically significant after controlling for body composition changes, suggesting that gut microbiota remodeling may have contributed, at least in part, to the observed metabolic benefits independently of body composition alterations. To the best of our knowledge, this is the first clinical investigation to elucidate the potential role of the ‘gut microbiota-intestinal barrier-inflammation’ axis in the therapeutic efficacy of Yijinjing exercise. Collectively, these results suggest that Yijinjing exercise, as a non-pharmacological approach associated with favorable gut microbiota adaptations and intestinal barrier reinforcement, may represent a promising strategy for T2DM management; however, the directionality and independence of the interrelated pathways between gut microbiota remodeling, body composition changes, and metabolic improvement warrant further investigation through larger-scale, mechanistically designed studies.

### 4.1 Interplay between gut dysbiosis, intestinal barrier integrity, and systemic inflammation in T2DM

Our findings reveal a significant reduction in gut microbial richness and diversity, accompanied by an overgrowth of pathobionts (e.g., Escherichia coli, Desulfovibrio, and Candida albicans) and a depletion of beneficial commensals (e.g., Lactobacillus and Bifidobacterium) in T2DM patients. These observations align with previous reports identifying gut dysbiosis as a potential environmental driver of T2DM (15, 16). However, the causal relationship between microbial shifts and T2DM remains complex. We propose that these interactions form a self-reinforcing vicious cycle rather than a unidirectional causal link. Hyperglycemia likely creates a favorable niche for the colonization of opportunistic pathogens and fungi (17). Our data demonstrate a significant positive correlation between fungal abundance, elevated C-reactive protein (CRP) levels, and markers of intestinal barrier dysfunction (D-LA and Zonulin). It is plausible that the increased fungal burden may promote systemic inflammation via the recognition of cell wall components (e.g., β-glucans) by innate immune receptors like Dectin-1, which subsequently may trigger a cascade of pro-inflammatory cytokines (18, 19). This systemic low-grade inflammation is a critical mediator of insulin resistance; for instance, inflammatory cytokines like TNF-α impair insulin signaling by promoting serine phosphorylation of the insulin receptor substrate-1 (IRS-1) (20). Furthermore, our study highlights a significant decrease in MFG-E8, a key protein for intestinal mucosal repair. Consistent with previous reports (21, 22), this impairment of mucosal integrity is consistent with the hypothesis that it facilitates the translocation of bacterial products into systemic circulation, thereby potentially amplifying the immune response. Collectively, these findings suggest that T2DM-associated hyperglycemia and dysbiosis synergistically disrupt the gut mucosal barrier. This disruption is associated with systemic inflammation, which in turn may exacerbates insulin resistance and metabolic dysfunction, thereby closing the loop of a “dysbiosis-barrier- inflammation” vicious cycle.

### 4.2 Regulatory effects of Yijinjing exercise on glucose homeostasis, systemic inflammation, and gut microbiota in T2DM

The selection of Yijinjing as the exercise modality in the present study was based on several physiological and mechanistic considerations that distinguish it from conventional exercise interventions. Unlike standard aerobic exercise programs such as brisk walking or resistance training, Yijinjing integrates three interdependent components: coordinated diaphragmatic breathing, meditative attentional focus, and sequential whole-body stretching involving sustained, low-to-moderate intensity isometric and isotonic contractions. This multimodal structure may confer physiological benefits that extend beyond those attributable to exercise intensity or energy expenditure alone.

First, the emphasis on diaphragmatic breathing in Yijinjing is particularly relevant to gut physiology. Slow, deep diaphragmatic breathing has been shown to enhance vagal tone and reduce sympathetic nervous system activity, which in turn may favorably modulate intestinal motility, mucosal immune function, and the gut microenvironment (23). This parasympathetic activation is distinct from the predominantly sympathetic activation associated with high-intensity conventional exercise, and may provide a more favorable milieu for the restoration of commensal microbial communities. Second, the meditative and attentional focus component of Yijinjing activates the hypothalamic-pituitary-adrenal (HPA) axis in a modulatory rather than stimulatory manner, potentially attenuating chronic cortisol-mediated immune dysregulation and intestinal barrier disruption that are commonly observed in T2DM patients under psychological stress (23, 24). Third, the sustained, sequential whole-body stretching movements of Yijinjing promote musculoskeletal flexibility and enhance peripheral blood circulation, including mesenteric perfusion, which may support intestinal mucosal repair - consistent with the MFG-E8 upregulation observed in the present study.

Nonetheless, we acknowledge that the present study did not include a parallel conventional exercise control group, and therefore cannot directly demonstrate that the observed effects are specific to Yijinjing rather than attributable to the general benefits of structured physical activity. The current findings should be interpreted as evidence that Yijinjing is an effective and feasible exercise modality for T2DM management, with mechanistic features that plausibly support its utility in targeting the gut-microbiota-host interface. Future randomized controlled trials directly comparing Yijinjing with conventional exercise programs of matched intensity and duration are necessary to formally establish the specificity of its effects.

Exercise is a cornerstone of T2DM management; however, the precise mechanisms by which it orchestrates multi-dimensional regulation through the “gut-muscle-immune” axis remain a subject of intense investigation (7, 25). Our results demonstrate that a 6-month Yijinjing intervention significantly ameliorates glucose homeostasis and insulin sensitivity in T2DM patients, concurrent with remodeling of the gut microbiome, reinforcement of the intestinal barrier, and suppression of systemic inflammation. Partial correlation analyzes further indicated that these gut microbiota changes were associated with improvements in insulin resistance and inflammatory markers independently of concurrent body composition changes, suggesting a potential contributory role of microbiota remodeling in the observed metabolic benefits.

Mechanistically, these clinical improvements likely arise from a synergistic integration of metabolic and immunological pathways. Exercise-induced metabolic remodeling plays a pivotal role; previous evidence suggests that physical activity alters the bile acid profile and enhances the production of short-chain fatty acids (SCFAs), such as butyrate (26–28). SCFAs serve not only as the primary energy source for colonocytes but also as potent activators of the AMPK pathway in skeletal muscle, thereby bridging the crosstalk between the gut microbiome and systemic glucose metabolism (29). Furthermore, as a mind-body practice integrating rhythmic breathing and deliberate stretching, Yijinjing may optimize the intestinal immune microenvironment—potentially by modulating intestinal blood flow and vagal tone. Our findings support this, as evidenced by the restoration of mucosal barrier integrity (reduced D-LA/Zonulin and elevated MFG-E8), which aligns with exercise-induced elevations in intestinal secretory IgA and the downregulation of pro-inflammatory cytokines such as IL-6 and TNF-α (30, 31). This restoration of immune homeostasis effectively hinders the colonization of pathobionts, thereby dismantling the “inflammation-barrier disruption” loop.

Finally, the exercise-induced optimization of body composition, characterized by increased lean mass and reduced adiposity, may also serve as an important modifier of the gut ecosystem (32). Given the complexity of directional causality between body composition changes, weight loss, and microbial shifts, the relative independent contributions of gut microbiota remodeling and body composition alterations to the observed metabolic improvements cannot be fully disentangled in the current study design. Nonetheless, our data support the notion that structured Yijinjing training is an effective strategy for optimizing the metabolic phenotype of T2DM patients. In conclusion, Yijinjing acts as more than a physical exercise; it serves as a therapeutic modality that is associated with favorable modulation of the gut-immune-metabolic axis. These findings highlight the potential of exercise-based microbiota adaptations as a promising avenue toward personalized, non- pharmacological interventions for T2DM, though mechanistically designed studies are needed to establish the causal hierarchy among gut microbiota remodeling, body composition changes, and metabolic improvement.

## 5 Limitations and future directions

Despite the novel insights provided by this study into the mechanisms underlying Yijinjing-mediated T2DM management, several limitations warrant consideration.

First, the single-arm pre-post design without a concurrent control group represents a fundamental methodological constraint. Although this design provides evidence of within-group change following the Yijinjing intervention, it precludes direct comparison with a sedentary control condition or an alternative exercise modality, and does not permit definitive establishment of causal relationships among the observed changes in gut microbial composition, intestinal barrier integrity, systemic inflammation, and glucose homeostasis. The causal hierarchy among these variables—for instance, whether gut microbiota remodeling preceded and drove improvements in intestinal barrier function and systemic inflammation, or whether these changes occurred in parallel as independent downstream effects—cannot be formally determined from the present data. Furthermore, the relative contribution of exercise per se versus the specific characteristics of Yijinjing—such as diaphragmatic breathing, meditative focus, and sustained musculotendinous stretching—cannot be formally disentangled. The mechanistic discussions presented in this manuscript should therefore be interpreted as biologically plausible hypotheses grounded in prior literature rather than causally demonstrated conclusions. Future randomized controlled trials (RCTs) incorporating a non-exercise control arm and an active comparator arm (e.g., brisk walking or Tai Chi of matched intensity and duration), with random allocation, allocation concealment, and blinded outcome assessment, are warranted to formally establish the specificity and efficacy of Yijinjing’s effects on the gut-microbiota-host interface in T2DM populations.

Second, the exclusive enrollment of male participants limits the generalizability of the findings to female T2DM populations. Given the well-documented influence of sex hormones—particularly estrogen and progesterone—on gut microbial composition, intestinal barrier integrity, and systemic inflammatory responses, the mechanistic associations reported here may not be directly extrapolated to women. Future studies should enroll both sexes with adequate sample sizes to permit sex-stratified analyzes, thereby elucidating whether the gut microbiota-remodeling effects of Yijinjing exercise are consistent across sexes or exhibit sex-specific patterns.

Third, potential confounding from dietary variability and medication use represents an important limitation. Although dietary adherence was monitored using structured 3-day dietary records (two weekdays and one weekend day) collected at multiple time points throughout the intervention, formal quantitative analysis of nutrient-level dietary intake—such as systematic comparison of daily energy intake, macronutrient distribution, and dietary fiber consumption across time points—was not performed. Residual dietary confounding therefore cannot be entirely excluded. Additionally, while all participants were maintained on stable oral hypoglycemic regimens and users of antibiotics, probiotics, and other microbiota-influencing agents were excluded at enrollment, the specific classes of oral hypoglycemic agents used by individual participants were not systematically recorded or stratified in the analyzes. Given that certain agents—most notably metformin—are known to independently modulate gut microbiota composition, future studies should incorporate formal nutrient-level dietary assessments and conduct stratified analyzes by medication class to more rigorously disentangle these confounding effects.

Fourth, the functional implications of the observed microbial shifts remain inferential in the absence of direct measurement data. The present study employed 16S rRNA amplicon sequencing, which provides taxonomic classification predominantly at the genus level and does not enable direct profiling of microbial functional capacity. Mechanistic pathway discussions—particularly those relating to SCFA production, vagal tone modulation, and LPS-mediated inflammatory signaling—were inferred from changes in microbial composition and supported by prior literature, rather than directly demonstrated. The absence of fecal SCFA quantification (e.g., by gas chromatography–mass spectrometry), serum bile acid profiling, heart rate variability (HRV) assessment, and intestinal permeability markers (e.g., serum LPS, zonulin) represents an important limitation. Future studies should integrate shotgun metagenomic sequencing, metatranscriptomics, and targeted metabolomics to formally substantiate the proposed mechanistic pathways.

Fifth, the 6-month follow-up period does not permit assessment of the long-term sustainability of the observed gut microbiota remodeling and metabolic improvements beyond the active intervention window. Given the dynamic sensitivity of gut microbiota to changes in physical activity and dietary behavior, it is plausible that the observed microbial shifts may partially regress following cessation of structured Yijinjing practice if adherence is not maintained. Future investigations should incorporate extended post-intervention follow-up assessments (e.g., at 6, 12, and 24 months post-intervention) to evaluate the durability of Yijinjing-induced changes and to identify the minimum effective maintenance dose required to sustain the observed metabolic and microbiota benefits.

## 6 Conclusion

This study demonstrates that T2DM is characterized by profound gut dysbiosis, including decreased microbial diversity, depletion of beneficial commensals, and impaired intestinal barrier integrity, all of which are intrinsically linked to systemic low-grade inflammation. A 6-month Yijinjing exercise intervention effectively ameliorates these pathological alterations by restoring gut microbial richness, reinforcing the mucosal barrier, and suppressing systemic inflammatory responses, ultimately leading to improved glucose homeostasis and insulin sensitivity. These findings provide a robust scientific rationale for the clinical application of Yijinjing as an adjunctive therapeutic strategy for T2DM, suggesting that targeted modulation of the gut-microbiota-host interface represents a promising frontier for personalized diabetes management.

## Data Availability

The raw data supporting the conclusions of this article will be made available by the authors, without undue reservation.
